# Patient Representation From Structured Electronic Medical Records Based on Embedding Technique: Development and Validation Study

**DOI:** 10.2196/19905

**Published:** 2021-07-23

**Authors:** Yanqun Huang, Ni Wang, Zhiqiang Zhang, Honglei Liu, Xiaolu Fei, Lan Wei, Hui Chen

**Affiliations:** 1 School of Biomedical Engineering Capital Medical University Beijing China; 2 Beijing Key Laboratory of Fundamental Research on Biomechanics in Clinical Application Capital Medical University Beijing China; 3 Information Center Xuanwu Hospital Capital Medical University Beijing China

**Keywords:** electronic medical records, Skip-gram, feature representation, patient representation, stroke

## Abstract

**Background:**

The secondary use of structured electronic medical record (sEMR) data has become a challenge due to the diversity, sparsity, and high dimensionality of the data representation. Constructing an effective representation for sEMR data is becoming more and more crucial for subsequent data applications.

**Objective:**

We aimed to apply the embedding technique used in the natural language processing domain for the sEMR data representation and to explore the feasibility and superiority of the embedding-based feature and patient representations in clinical application.

**Methods:**

The entire training corpus consisted of records of 104,752 hospitalized patients with 13,757 medical concepts of disease diagnoses, physical examinations and procedures, laboratory tests, medications, etc. Each medical concept was embedded into a 200-dimensional real number vector using the Skip-gram algorithm with some adaptive changes from shuffling the medical concepts in a record 20 times. The average of vectors for all medical concepts in a patient record represented the patient. For embedding-based feature representation evaluation, we used the cosine similarities among the medical concept vectors to capture the latent clinical associations among the medical concepts. We further conducted a clustering analysis on stroke patients to evaluate and compare the embedding-based patient representations. The Hopkins statistic, Silhouette index (SI), and Davies-Bouldin index were used for the unsupervised evaluation, and the precision, recall, and F1 score were used for the supervised evaluation.

**Results:**

The dimension of patient representation was reduced from 13,757 to 200 using the embedding-based representation. The average cosine similarity of the selected disease (subarachnoid hemorrhage) and its 15 clinically relevant medical concepts was 0.973. Stroke patients were clustered into two clusters with the highest SI (0.852). Clustering analyses conducted on patients with the embedding representations showed higher applicability (Hopkins statistic 0.931), higher aggregation (SI 0.862), and lower dispersion (Davies-Bouldin index 0.551) than those conducted on patients with reference representation methods. The clustering solutions for patients with the embedding-based representation achieved the highest F1 scores of 0.944 and 0.717 for two clusters.

**Conclusions:**

The feature-level embedding-based representations can reflect the potential clinical associations among medical concepts effectively. The patient-level embedding-based representation is easy to use as continuous input to standard machine learning algorithms and can bring performance improvements. It is expected that the embedding-based representation will be helpful in a wide range of secondary uses of sEMR data.

## Introduction

The past decade has witnessed an explosion in the amount of digital information stored in electronic medical records (EMRs), which contain massive quantities of information on the clinical history of patients. The wide secondary use of this information for various clinical applications has become a prevalent trend [1], helping to make diagnostic decisions [2-4], predict patient outcomes [5-8], and provide treatment recommendations [9-11].

As we all know, the method of data representation is becoming more and more crucial for the performance of data applications [12,13]. Recently, many researchers have made preliminary attempts to convert different types of medical data to vectors by representation learning. They have then applied EMR data with these representations to clinical tasks [6,14,15], making more effective use of medical data and improving performance in the predictive analyses. Cui et al [6] compared the performances of three distributed representation methods (ie, Skip-gram, Continuous Bag-of-Words, and latent semantic analysis) for the prediction of hospital cost and length of stay (LOS). Ning et al [15] trained vector representations for medical concepts from biomedical journal articles through Skip-gram and proposed a fully automated feature extraction method for disease phenotyping based on the medical concept vector representation. Moreover, some researchers learned patient representation through deep learning [3,5,12]. Zhe Wang et al [5] designed a feature rearrangement representation based on the convolutional neural network for heart failure mortality prediction. Lei Wang et al [3] used autoencoder, an unsupervised deep learning algorithm, to generate lower-dimensional representations from EMR data in various predictive tasks such as readmission prediction and pneumonia prediction. A similar study [12] used the recurrent neural network–based denoising autoencoder to encode patient records into low-dimensional and dense vectors for heart failure prediction.

However, there are still challenges in the representation of structured EMR (sEMR) data containing high-dimensional and diverse features. Such features as demographic characteristics, disease diagnoses, physical examinations and procedures, and laboratory tests may have discrete or continuous values, making it difficult to reveal the latent relations among them. Moreover, it is difficult to make full use of every available feature (laboratory tests, for example) due to the unavoidable missing values. It is worth exploring how to deal with the patient records with features that are unequal in length.

Therefore, in this study, we leveraged a distributed embedding technique originated in natural language processing (NLP), the Skip-gram algorithm, with several adaptive changes to obtain effective representations from the sEMR data. The feature representation was evaluated by the dimension reduction visualization method and feature correlation analysis method. We further conducted clustering analyses on patients expressed with the proposed representations to evaluate the representation scheme. We aimed to explore the feasibility and superiority of the embedding-based representations in data mining tasks for sEMR data.

## Methods

### Study Data and Data Preprocessing

The sEMR data of 144,375 hospital admissions for 104,752 patients were collected from Xuanwu Hospital, Capital Medical University, Beijing, China, between January 2014 and December 2016. Patients’ features were grouped into seven major categories: demographic characteristics, hospital admission and discharge, utilization of medical resources, disease diagnoses (identified by International Classification of Disease, Tenth Revision [ICD-10] code), examination and procedures undergone (identified by International Classification of Diseases, Ninth Revision, Clinical Modification [ICD-9-CM] code), laboratory tests, and medications (Table S1 in Multimedia Appendix 1). They were maintained for each hospital stay. If a patient had multiple hospitalizations or multiple laboratory tests, only the first hospitalization or laboratory test was included. Patients’ personal information was completely removed from the data set before we could access the data remotely, ensuring the data were used in an anonymous and safe manner. The study and data use were approved by the Human Research Ethics Committees of the hospital.

Data analysis concentrated on a certain disease would be more targeted and specific because a certain group of patients may have similar characteristics. Stroke is a severe disease with high prevalence, high mortality, and high disability [16,17]. It is meaningful and crucial to mine the knowledge hidden in the data for stroke diagnosis and treatment. Thus, we focused on stroke patients for representation evaluation. In the data set, there were 8232 records involving adult patients with a primary diagnosis of stroke (ICD-10 codes I60 to I64, I66, and I67.8 [18]). Among them, 1397 patients had a primary diagnosis of hemorrhagic stroke (HS; ICD-10 codes I60 to I62) and 6835 of ischemic stroke (IS; ICD-10 codes I63, I64, I66, and I67.8).

Because the Skip-gram algorithm required discrete inputs, values of continuous features were binned into several discrete values. Age was grouped into <18, 18-34, 35-44, 45-59, and ≥60 years. Each laboratory test item was categorized into 2 classes (normal and abnormal) or 3 classes (high, medium, and low) according to the clinical laboratory test references. Other continuous features were grouped into 4 percentile bins (quartiles), each containing one-fourth of all samples (Table S1 in Multimedia Appendix 1). Therefore, a feature had several discrete values called medical concepts. For example, the feature “sex” had two concepts, male and female. If a patient record was considered as a sentence, the medical concepts in the record were then considered as words in the sentence. All the records composed the training corpus. Features involved in the representation included demographic characteristics, hospital admission, utilization of medical resources, disease diagnoses, physical examinations and procedures, laboratory tests, and medications. Features related to the patient’s outcomes, including LOS, hospital cost, and the discharging route, were used to evaluate the patient representations; thus, they were excluded from both training corpora. The full corpus consisted of 13,757 unique medical concepts derived from 104,752 patient records, while a subcorpus consisted of 3769 unique medical concepts derived from records of 8232 stroke patients.

Medical concepts were initially encoded in one-hot vectors, where the dimension of the one-hot vector equaled the number of distinct concepts in the data set. In the one-hot code scheme, a vocabulary of all the distinct medical concepts in the corpus was generated first; then, each medical concept was represented as a 0-1 vector, where the index of the target concept in the vocabulary was set to 1, and all the others were set to 0.

### Embedding-Based Representation

We used the Skip-gram algorithm [19] to learn the representation of medical features. The Skip-gram algorithm can map words into a low-dimensional real number space where the relevant words were located closely. Assuming that similar words may share similar contexts, the Skip-gram algorithm predicted surrounding words of the current (target) word. The same context prediction was repeated as the target word moving to the next. The goal of the Skip-gram algorithm was to maximize the following average log probability:


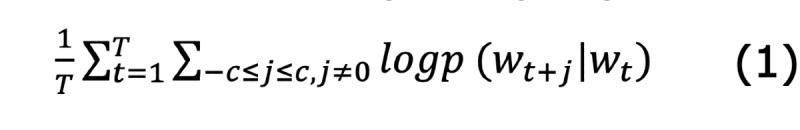



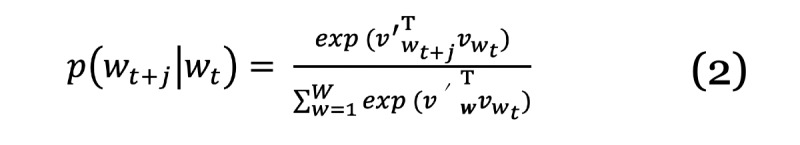


where *T* was the length of the sentence that contained the target word, *c* (set to 5 in this study) was the size of the training context (called window size), *w_t_* and *w_t+j_* denoted the target word and the *j*th neighboring words before or after the target word in the training context window, *v* represented the *d*-dimensional (*d* was set to 200 in this study) real number vector of the word, and *W* (13,757 and 3769 for full corpus and stroke corpus in this study) was the total number of words in the corpus.

Unlike a natural language sentence with a relatively fixed word order, a medical concept’s location in a record was appointed manually. It was difficult to assume that the more relevant the concepts were, the closer they were located in a record. Therefore, medical concepts relevant to the target concept might not appear in the training context window in Equation (1). To reduce the impact of the concept sequence on the Skip-gram algorithm, we used the shuffling mechanism [14] to rearrange the order of medical concepts within each record in the corpus randomly. The shuffled corpus was then used for training embedding vectors. The shuffling-training process was performed 20 times, resulting in 20 embedding vectors corresponding to one medical concept. The average of these vectors was considered the final embedding representation of the concept. Because a patient could only take one medical concept for a certain feature, the feature was therefore represented as an embedding vector. After training with the Skip-gram algorithm, a patient who had *k* medical concepts for *k* features would have *k* real number vectors. The average of these vectors was considered the embedding-based representation for the patient.

### Evaluation of the Representation Schemes

#### Evaluation of the Feature Representation

The feature representation was first evaluated visually by mapping the d-dimensional real number vector space into a two-dimensional space using the t-distributed stochastic neighbor embedding (t-SNE) algorithm [14,20]. We used the software Python 3.7 and the sklearn.manifold.TSNE tool for the visualization. The t-SNE algorithm’s main parameters were as follows: dimension of the embedded space=2, perplexity=30, learning rate=200, number of iterations=1000, gradient calculation method=Barnes-Hut, and angle=0.5. We compared the reduction visualization of medical concepts’ vectors training with different corpora. For the purpose of clarity, 441 diagnosis concepts that occurred in at least ten records in the stroke corpus were mapped into the two-dimensional space. They were divided into 14 categories according to the Clinical Classifications Software code [21] for further analysis.

The embedding-based feature representation was then evaluated on how it could capture the latent association among features. We identified the 10 closest medical concepts from each of the diagnosis, laboratory test, physical examination and procedure, medication, and other feature categories in the low-dimensional embedding space for two index diagnosis concepts: subarachnoid hemorrhage (SAH) and occlusion and stenosis of middle cerebral artery (OSMCA). The similarities between the index diagnosis concepts and others were measured by cosine similarity, which was suitable for numerical vectors.

#### Evaluation of the Patient Representation

The distributed embedding technique had the advantage of revealing the potential relevance among samples [19], and the unsupervised clustering analysis was a machine learning task that depended more on the sample relevance. Therefore, clustering analysis was used for determining whether the proposed patient representation had a certain advantage in revealing the potential relevance among patients, thus making the clustering solution more aggregative. For the purpose of comparison, 6 embedding-based patient representation schemes and 2 reference schemes were employed. Four embedding-based representations were generated using the initial full corpus, the initial stroke corpus, the shuffled full corpora, and the shuffled stroke corpora as the training corpus. Additionally, to explore the impacts of the numbers of features included in the training context on the representations, we also designed two representation learning schemes that used the initial full and stroke corpora with the maximum window sizes of 255 and 224, respectively. The maximum window size was the length of the record that had the most medical concepts in the corpus. Two commonly used data representation methods were used as the reference methods; one was the multi-hot representation, which was the bitwise summations of one-hot codes for all features, and the other was the mixture of multi-hot codes for discrete features and original values for continuous features. In the mixture representation, we selected 59 laboratory tests in at least 90% of stroke patients. Missing values in the laboratory tests were interpolated using the median of the corresponding laboratory tests. Figure S1 in Multimedia Appendix 1 depicts the representation schemes used in this study with simple examples.

We conducted k-means clustering analyses on the stroke patients, using cosine distance for the embedding-based representations, Jaccard distance [22] for the multi-hot representation, and Jaccard distance (for discrete features) plus cosine distance (for continuous features) for the mixture representation. We evaluated clustering solutions by Hopkins statistics [23], Silhouette index (SI) [24], and Davies-Bouldin index (DBI) [24]. Hopkins statistics describe the uniformity of data for clustering, while the SI validates the consistency within clusters, and the DBI measures the average similarity between each cluster and the one that most resembles it. The values of Hopkins statistics and DBI range from 0 to 1, while the value of SI ranges from −1 to 1. Higher Hopkins statistics and SI and lower DBI suggest better clustering results. SI was also used to compare k-means clustering solutions for different values of k to determine the optimal number of clusters in this study. The features related to the patient’s outcomes were compared to identify the differences between the clusters. Clustering solutions were also assessed concerning the clinical characteristics, including demographic characteristics, utilization of medical resources, disease diagnoses, laboratory tests, procedures, and patient outcomes. Differences in these clinical features were compared among clusters by statistical tests, aiming to confirm whether the knowledge discovered by the clustering analyses was consistent with the clinical facts or new to the medical domain.

## Results

### Feature Representation Visualization

**Figure 1:**
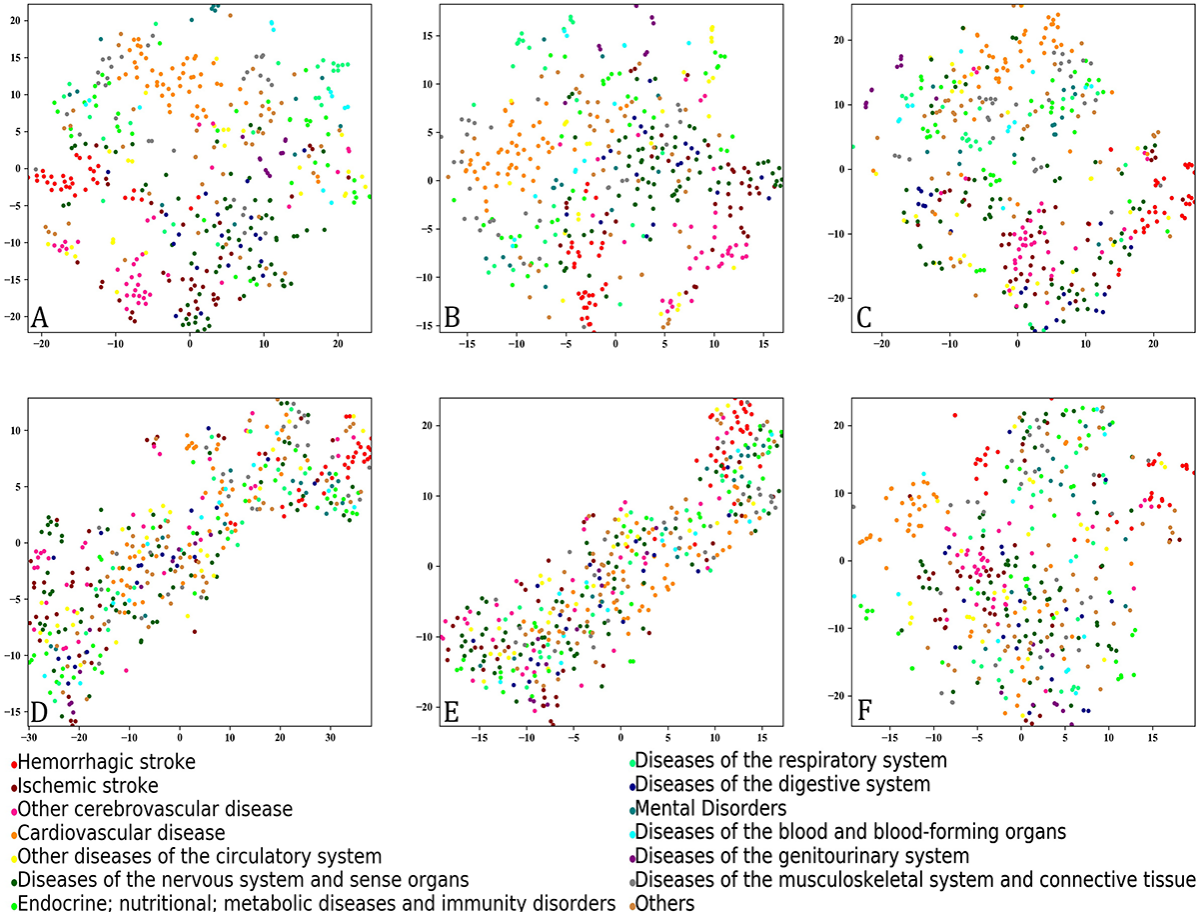
Embedding vectors of diagnosis concepts in the t-distributed stochastic neighbor embedding space. The embedding vectors were trained by Skip-gram algorithm with a window size of 5 from (A) the shuffled full corpora, (B) the initial full corpus, (D) the shuffled stroke corpora, and (E) the initial stroke corpus, with a window size of 255 from (C) the initial full corpus, and with a window size of 224 from (F) the initial stroke corpus.

Figure 1 shows the embedding vectors for disease concepts trained with different corpora in the two-dimension space. Vectors for disease concepts trained with the stroke corpus (Figures 1D and E) were more concentrated than those with the full corpus (Figures 1A and B). Further, disease vectors trained with the shuffled full corpus (Figure 1A) showed stronger disease aggregation compared with those trained with the initial full corpus (Figure 1B) and those trained with the full corpus using the maximum window size (Figure 1C).

**Figure 2:**
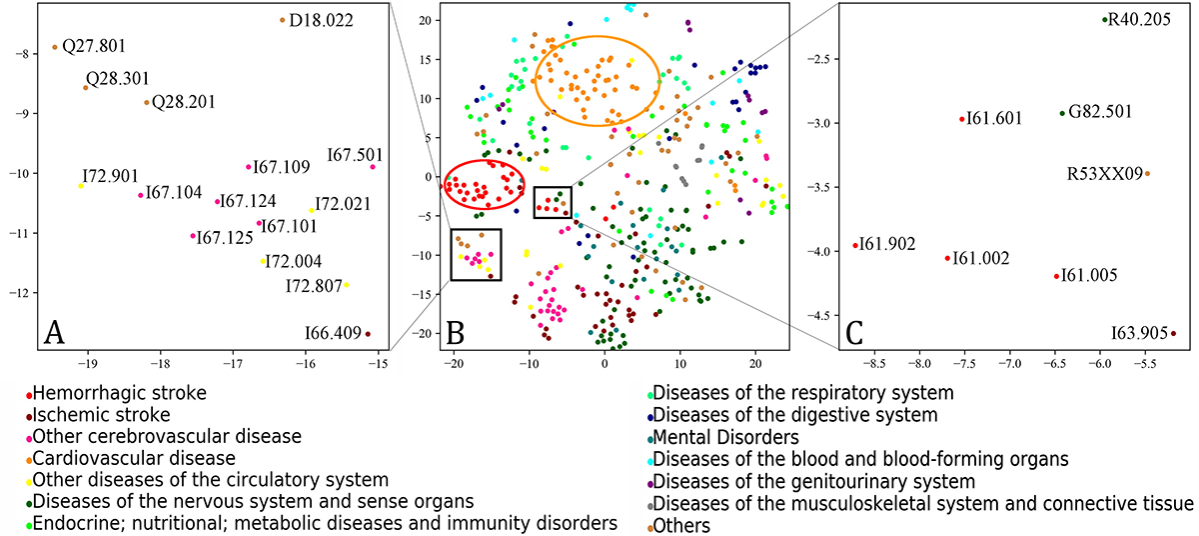
Visualization of the selected 441 diagnosis concepts in the embedding space. (A) and (C) are the locally enlarged areas in black rectangle boxes of (B), in which the embedding vectors were trained from the full corpus with the concept shuffling and were projected to a place by the t-distributed stochastic neighbor embedding technique.

As shown in Figure 2B, most of the disease concepts related to hemorrhagic stroke (red dots) and cardiovascular disease (orange dots) were concentrated inside the red and orange circles, respectively, suggesting that disease concepts of the same categories were more likely to come together in the embedding space. Further, the nearby medical concepts in the embedding space were usually clinically correlated. For example, in the embedding space, the disease concepts coded by different ICD-10 codes but in the same rough disease category were able to gather together (eg, circulatory system disease with ICD-10 codes beginning with Q2 and I in Figure 2A). Additionally, as shown in Figure 2C, diseases of abnormal perception symptoms and signs such as coma (ICD-10 code, R40.205), tetraplegia (G82.501), and malaise and fatigue (R53xx09) were adjacent to cerebrovascular diseases of intracerebral hemorrhage (I61.002, I61.005, I61.601, and I61.902) and cerebral infarction (I63.905). This was consistent with the clinical facts [25].

### Features Correlation Analysis

Table 1 lists the 3 closest medical concepts (from different feature categories) to two cerebrovascular diseases: SAH and OSMCA. Among medical concepts of different categories, even if heterogeneous, clinically relevant concepts could be identified by the cosine similarity among concept vectors. For example, the closest laboratory tests to SAH were red and turbid cerebrospinal fluid, consistent with clinical fact. Moreover, the embedding vectors could reveal more detailed information about the medical concepts in the same rough category. For two diagnosis concepts of typical cerebrovascular diseases, SAH and OSMCA, the closest procedures were aneurysm clipping and percutaneous drug-eluting stent implantation, which were usually used for treating SAH and OSMCA in clinical, respectively. Besides, the closest concepts to the same index concept were not precisely the same when their representations were training with the full and stroke corpus, but both were clinically relevant to the index concept. We also noticed that the cosine similarities between the index concept and their closest concepts in the stoke corpus were larger than in the full corpus. For example, the average of the similarities of the disease SAH and its 15 closest medical concepts were 0.910 and 0.973 in the full and the stroke corpus, respectively. Table S2 in Multimedia Appendix 1 shows the 10 medical concepts closest to SAH and OSMCA for each feature category.

**Table 1:** The 15 closest medical concepts whose embedding representations were trained with the full corpus and the stroke corpus of the disease concepts subarachnoid hemorrhage and the occlusion and stenosis of middle cerebral artery.

Category	Occlusion and stenosis of middle cerebral artery			Subarachnoid hemorrhage			
	Closest concept^a^	Similarity	Closest concept^a^		Similarity	Closest concept^b^	Similarity
Disease diagnoses	Occlusion and stenosis of anterior cerebral artery	0.964	Subarachnoid hemorrhage from anterior communicating artery		0.932	Subarachnoid hemorrhage from posterior communicating artery	0.976
	Occlusion and stenosis of multiple and bilateral cerebral arteries	0.962	Subarachnoid hemorrhage from posterior communicating artery		0.929	Subarachnoid hemorrhage from anterior communicating artery	0.975
	Occlusion and stenosis of posterior cerebral artery	0.958	Bronchitis, not specified as acute or chronic		0.925	Aneurysm	0.971
Laboratory tests	Platelet aggregation test with turbidimetry: high	0.915	Cerebrospinal fluid color: red		0.933	Cerebrospinal fluid transparency: turbid	0.975
	Plasma protein C: high	0.914	Cerebrospinal fluid transparency: turbid		0.904	Cerebrospinal fluid color: blood color	0.959
	Platelet aggregation test with turbidimetry: low	0.910	Cerebrospinal fluid color: orange		0.863	White blood cell count in cerebrospinal fluid: high	0.958
Procedures	Percutaneous drug-eluting stent implantation	0.861	Embolization of intracranial aneurysm		0.985	Embolization of intracranial aneurysm	0.986
	Percutaneous drug-eluting stent implantation of subclavian artery	0.848	Aneurysm clipping		0.974	Aneurysm clipping	0.974
	Transcranial angioplasty	0.822	Embolization of intracranial vessels		0.960	Skull titanium plate placement	0.965
Medications	Probucol tablet	0.938	Hypertonic sodium chloride hydroxyethyl starch 40 injection		0.938	Tramadol	0.987
	Songling Xuemaikang capsule^c^	0.924	Nimodipine		0.895	Fasudil	0.983
	Yufeng Ningxin Drop Pills^c^	0.920	Fructose sodium diphosphate injection		0.894	Dezocine injection	0.982
Others	Allergic to metformin	0.858	Neurosurgery ICU^d^		0.924	ICU of Neurosurgery department	0.976
	Allergic to vinpocetine	0.852	Ventilator utilization		0.796	Discharge department: Neurosurgery department	0.964
	Allergic to iopromide	0.852	Discharge department: Neurosurgery department		0.796	Admission department: Neurosurgery department	0.962

^a^Embedding vectors of concepts were trained with the full corpora.

^b^Embedding vectors of concepts were trained with the stroke corpora.

^c^Traditional Chinese medication.

^d^ICU: intensive care unit.

### Patient Clustering Analysis

In the k-means clustering analyses, the optimal k was determined to be 2, where the corresponding SI value was the highest when k changed from 2 to 15 in each of the representation schemes (Figure S2 in Multimedia Appendix 1). The greatest values of Hopkins statistics (0.931) and SI (0.862) and the lowest value of DBI (0.551) were seen in the clustering solution in which patients were represented by the embedding vectors (Table 2), suggesting that patients with the embedding vectors could be clustered with higher uniformity and aggregation and lower dispersion.

**Table 2:** Clustering performance on interval evaluation indexes based on various patient representations.

Representation schemes	Parameters for training				Cluster evaluation indexes		
	Corpus used	Corpus with shuffling	Window size	Hopkins statistic		Silhouette index	Davies-Bouldin index
Embedding-based representation	Full	Yes	5	0.922		0.783	1.067
	Stroke	Yes	5	0.913		0.862^a^	0.551^b^
	Full	No	5	0.903		0.685	1.711
	Stroke	No	5	0.925		0.672	1.382
	Full	No	255	0.922		0.783	1.065
	Stroke	No	224	0.931^c^		0.790	0.772
Multi-hot representation^d^	N/A^e^	N/A	N/A	0.813		0.233	3.236
Mixture representation^f^	N/A	N/A	N/A	0.918		0.141	4.157

^a^Highest value of the Silhouette index.

^b^Lowest value of the Davies-Bouldin index.

^c^Highest value of the Hopkins statistic.

^d^Multi-hot representation: representation method of the combinations of one-hot codes.

^e^N/A: not applicable.

^f^Mixture representation: representation method of the combination of multi-hot codes for discrete features and real numbers for continuous values of age and laboratory tests.

Among the 8 clustering solutions, cluster 1 contained an average of 6869 (range 6214-7704) patients, of whom 92.2% (range 85.5%-95.7%) had a primary diagnosis of IS. Cluster 2 contained an average of 1363 (range 528-2018) patients, of whom 63.1% (range 51.2%-74.5%) had a primary diagnosis of HS. Therefore, we used IS as the label of patients in cluster 1 and HS as the label of patients in cluster 2. Among the embedding-based representations, the representation trained with the shuffled full corpus reached the greatest F1 scores of 0.944 and 0.717 for clusters 1 and 2, respectively (Table 3). In this clustering solution, 95.0% (6495/6835) of the IS patients and 69.4% (970/1397) of the HS patients were correctly grouped into clusters 1 and 2, respectively. Among the patients (340/6835, 5.0%) with a primary diagnosis of IS who were grouped into cluster 2, 9.4% (32/340) of them had HS as the secondary diagnosis. Meanwhile, among the patients (427/1397, 30.6%) with a primary diagnosis of HS who were grouped into cluster 1, 48.9% (209/427) of them had IS as the secondary diagnosis. In this situation, the clustering performance might be underestimated.

Between clusters 1 and 2 of stroke patients represented by the embedding vectors learned from shuffled full corpus, there were significant differences in mortality rate (45/6922, 0.65% vs 91/1310, 6.95%, *P*<.001), cost per hospital stay (17.7 vs 113.0 thousand yuan renminbi, *P*<.001), and LOS (9.8 vs 12.6 days, *P*<.001). Patients in cluster 2 occupied more medical resources than those in cluster 1 concerning the ventilator (544/1310, 41.5% vs 105/6922, 1.5%, *P*<.001) and intensive care unit (1025/1310, 78.2% vs 353/6922, 5.1%, *P*<.001). This might partially be linked to the fact that patients in cluster 2 usually also had such acute diseases as pneumonia (189/1310, 14.4% vs 318/6922, 4.6%, *P*<.001), while patients in cluster 1 had chronic diseases like paralysis (3735/6922, .54.0% vs 119/1310, 9.1%, *P*<.001). Table S3 in Multimedia Appendix 1 depicts more comparisons.

**Table 3:** Clustering performance on interval evaluation indexes based on various patient representations.

Representation	Parameters for training				True label		Cluster 1 patients, n		Cluster 2 patients, n		Evaluation indexes			
	Corpus used	Shuffle	Window size							Precision		Recall	F1 score	
Embedding-based	Full	Yes	5	IS^a^		6495		340		0.938		0.950	0.944^b^	
				HS^c^		427		970		0.740		0.694	0.717^d^	
	Stroke	Yes	5	IS		6530		305		0.928		0.955	0.942	
				HS		506		891		0.745		0.638	0.687	
	Full	No	5	IS		6587		248		0.855		0.964	0.906	
				HS		1117		280		0.530		0.200	0.291	
	Stroke	No	5	IS		6472		363		0.903		0.947	0.924	
				HS		699		698		0.658		0.500	0.568	
	Full	No	255	IS		6305		530		0.927		0.922	0.925	
				HS		493		904		0.630		0.647	0.639	
	Stroke	No	224	IS		6378		457		0.932		0.933	0.932	
				HS		467		930		0.671		0.666	0.668	
Multi-hot^e^	N/A^f^	N/A	N/A	IS		5874		961		0.938		0.859	0.897	
	N/A	N/A	N/A	HS		388		1009		0.512		0.722	0.599	
Mixture^g^	N/A	N/A	N/A	IS		5945		890		0.957		0.870	0.911	
	N/A	N/A	N/A	HS		269		1128		0.559		0.807	0.661	

^a^IS: ischemic stroke.

^b^Highest F1 score for cluster 1.

^c^HS: hemorrhagic stroke.

^d^Highest F1 score for cluster 2.

^e^Multi-hot: representation method of the combinations of one-hot codes.

^f^N/A: not applicable.

^g^Mixture: representation method of the combination of multi-hot codes for discrete features and real numbers for continuous values of age and laboratory tests.

## Discussion

### Principal Findings

Representation for structured medical data is critical for data mining tasks in the medical domain [3,5,6,14]. The one-hot code scheme is a simple and widely used representation. However, it may be unsuitable for the complex and diverse EMR data due to its high dimensionality and sparsity. Analyses of massive one-hot coded data may require greater computational power because of not only their high-dimensional and sparse nature but also the unclear potential relevance of the data [26]. Therefore, many studies have focused on effective and efficient data representation. In this study, we adopted an embedding-based method derived from NLP techniques to represent the structured patient data. The proposed representations brought a deep and intuitive insight into associations among medical concepts and a great performance improvement in a similarity-based data mining task.

The distributed embedding representations have the merits of low dimensionality and the capability for revealing the latent relationship among the represented objects [19]. Thus, the embedding-based or deep learning–based representation has been widely used in various applications, especially in the clinical NLP domains, to represent unstructured medical texts, including biomedical publications [27], clinical notes [28], and radiology reports [29-31]. With these representations, researchers could perform feature engineering with less expert effort and transform raw texts into low-dimensional dense vectors with clinical meanings and further identify implicit patterns in patients. Inspired by the representation learning from the unstructured medical data, researchers adopted these representation methods for structured medical data, including medical codes such as diagnosis codes, procedure codes, and drug codes [5,32], laboratory tests [12], and time-related data, which was informative for patients [1,12,33,34].

In this study, we borrowed the idea from this originally text-oriented technique and applied it to sEMR data with diverse patient features. We embedded each medical concept into a low-dimensional real number vector using the Skip-gram algorithm. Both the visualized and quantitative analyses showed that the embedding-based feature representation provided a relatively clear understanding of the associations and connections among the medical concepts, which were consistent with medical knowledge and clinical practice. On the other hand, clustering solutions on patients represented with embedding vectors showed a better clustering nature than those expressed with multi-hot vectors. The embedding-based representation showed advantages in dimension reduction and in the convenience of numerical computation and association mining in this study.

An informative representation was usually derived from different modalities and medical data sources, such as cross-sectional and longitudinal data, and quantitative indexes and narrative notes. In this study, demographic characteristics, diagnoses, physical examinations and procedures, laboratory tests, medications, and hospital admission and discharge were all brought into the feature representation learning. A particular and unavoidable characteristic of laboratory tests was that patients might take different laboratory test items according to the need for diagnosis and treatment. This must result in lots of missing values for laboratory tests. The joint use of the discretization of continuous values and the Skip-gram algorithm solved the problem, making all the available features to be fully used. Clustering analyses showed that patients represented by embedding vectors were more likely to cluster together than those represented in the original form, where about three-quarters of laboratory tests were dropped due to missing values. It may partially attribute to the inclusion of all the features and discretization of the continuous features.

When using the Skip-gram algorithm for representation learning for sEMR data, several adaptive changes had been made. First, we applied the shuffling mechanism when building the training corpus to reduce the impact of the concept order on the coverage of the training context. Glicksberg et al [14] randomly shuffled the medical concepts within a time interval. We further extended the idea of shuffling concepts. The medical concepts were rearranged randomly within a patient record 20 times. The resulting 20 embedded vectors for each medical concept trained with different shuffled corpora were then averaged as the final embedding vector. Results from several evaluation tasks showed that the shuffling-based representation at both the feature and patient level had a more satisfactory performance compared with their not shuffling-based counterparts.

In the Skip-gram algorithm, the range of training context was also crucial to the algorithm performance. For the same reason as for using a shuffling method, we set the window size to the maximum to have the training context covered the most neighboring concepts. However, there was no outstanding performance improvement in the clustering task. The finding was consistent with other studies [33,35] that found performances got worse as the window size increased. It indicated that wide training context might introduce redundant information or even noise to the training. Besides, the corpus used in the Skip-gram algorithm was also linked to the performance improvement on the clustering task in this study. Stroke patients whose representations were trained with the corpus including all the patient records were clustered into two groups with higher aggregation and lower dispersion than those whose representations were trained with the corpus including only stroke patients’ records. The finding was similar to that of a study by Yanshan Wang et al [27] that the embedding-based representation from the public domain corpora showed more satisfactory results in biomedical information retrieval than from the biomedical domain corpora.

### Limitations

Our study had some limitations. First, we did not use time-oriented patient records with critical importance for evaluating the patient course and prognosis. The history of medical events may affect future medical events; these medical sequence data are crucial for clinical diagnosis and treatment. Rich time-oriented data, including time-series features in an inpatient record and the temporality between multiple inpatient records, were used for learning patient representations by some algorithms targeted at sequence data, such as recurrent neural network [12], time-aware attention method [33], Deepr [36], and Patient2Vec [37]. Those time-related representations, which captured patients’ sequential information from a longitudinal perspective, could be used for supervised prediction tasks [12,36,37], and the unsupervised task-like disease clustering analyses at the feature level [33]. In contrast, we just took the cross-sectional data with diverse feature types of patients, focusing on a certain hospitalization’s static characteristics, into the effective representations by Skip-gram algorithm at both feature and patient levels. Second, a patient representation was just a simple average of the embedding vectors for features with equal weights. This may be not completely consistent with the fact that clinical features may have different importance to the diagnosis of a specific disease. Last, we only evaluated the effectiveness of the embedding-based patient representation with clustering analysis. The proposed patient representation needs more validation in various clinically meaningful tasks.

### Conclusions

In this study, we applied an embedding technique in learning the patient representation from sEMR data with different types of clinical features. With the original Skip-gram algorithm’s adaptive changes, the embedding-based representations could somehow reflect the potential associations among features and patients. The performance improvement in a clinically meaningful clustering task suggested the proposed patient representation’s effectiveness and efficiency. It is expected that the embedding-based representation will be helpful in a wide range of secondary uses of EMR data.

## Appendix

Multimedia Appendix 1 Supplementary materials.

## Abbreviations

DBI: Davies-Bouldin index
EMR: electronic medical record
HS: hemorrhagic stroke
ICD-10: International Classification of Diseases, Tenth Revision
ICD-9-CM: International Classification of Diseases, Ninth Revision, Clinical Modification
IS: ischemic stroke
LOS: length of stay
NLP: natural language processing
OSMCA: occlusion and stenosis of middle cerebral artery
SAH: subarachnoid hemorrhage
sEMR: structured electronic medical record
SI: Silhouette index
t-SNE: t-distributed stochastic neighbor embedding
