# Supplementary material for: Patient Representation From Structured Electronic Medical Records Based on Embedding Technique: Development and Validation Study
Source: JMIR Med Inform. 2021 Jul 23;9(7):e19905. doi: 10.2196/19905 (PMC8367145; doi:10.2196/19905)

## Table S1. Patient features and corresponding medical concepts in the dataset.

| Feature category | Feature | Concept | # of concept in the full corpus | # of concept in the stroke corpus |
| --- | --- | --- | --- | --- |
| Demographic characteristics | Age (years) | <18, 18–34, 35–44, 45–59, and ≥60 | 5 | 4 |
|  | Sex | Male and female | 2 | 2 |
|  | Marital status | Unmarried, married, divorced, and widowed | 4 | 4 |
|  | Occupation | Civil servants, workers, students, etc. | 13 | 11 |
|  | Nationality | Han, Hui, Mongolian, etc. | 37 | 16 |
|  | Blood type | A, B, AB, O, Rh-negative and Rh-positive | 6 | 6 |
|  | Allergen | Penicillin, seafood, pollen, etc. | 634 | 147 |
|  | Medical insurance status | Urban employee basic medical insurance, urban residents’ basic medical insurance, etc. | 6 | 6 |
| Hospital admission | Source of admission | Clinic, emergency department, transfer, and other | 4 | 2 |
|  | Department at admission | Neurology department, endocrinology department, etc. | 25 | 19 |
|  | Department at discharge | Respiratory department, neurosurgery department, etc. | 25 | 15 |
|  | Length of stay (days) | <4, 4-6, 7-11, and >11 | 4 | 4 |
|  | Cost (RMB yuan) | <5638, 5638-11488, 114898-26132, and >26132 | 4 | 4 |
|  | Readmission | Yes and no | 2 | 2 |
| Utilization of medical resources | Ventilator | Yes | 1 | 1 |
|  | Intensive care unit (ICU) | Neurosurgery ICU, neurology ICU, etc. | 17 | 9 |
| Disease diagnoses | Diagnoses | subarachnoid hemorrhage, Type 2 diabetes mellitus, etc. | 8692 | 1901 |
| Physical examinations and Procedures | Procedures | Echocardiography, aneurysm clipping, Transcranial angioplasty, etc. | 2537 | 369 |
| Laboratory tests | Test items | Normal and abnormal, or high, medium, and low | 718 | 548 |
| Medications | Medication | Amoxicillin, aspirin, etc. | 1031 | 709 |
| Discharge deposition | Discharge deposition | Discharge under doctor’s advice, death, etc. | 6 | 6 |

## Table S2. Fifty closest medical concepts whose embedding vectors learned with the full corpus and the stroke corpus of the disease concepts occlusion and stenosis of middle cerebral artery and subarachnoid hemorrhage.

|  | Occlusion and stenosis of middle cerebral artery | Subarachnoid hemorrhage | |
| --- | --- | --- | --- |
|  | **Closest concept^a^- similarity** | **Closest concept^a^- similarity** | **Closest concept^a^- similarity** |
| Disease diagnoses | Subarachnoid hemorrhage from anterior communicating artery-0.932 | Occlusion and stenosis of anterior cerebral artery-0.964 | Subarachnoid hemorrhage from posterior communicating artery-0.976 |
|  | Subarachnoid hemorrhage from posterior communicating artery-0.929 | Occlusion and stenosis of multiple and bilateral cerebral arteries-0.962 | Subarachnoid hemorrhage from anterior communicating artery-0.975 |
|  | Bronchitis, not specified as acute or chronic-0.925 | Occlusion and stenosis of posterior cerebral artery-0.958 | Aneurysm-0.971 |
|  | Subarachnoid hemorrhage from middle cerebral artery-0.911 | Occlusion and stenosis of carotid artery-0.948 | Subarachnoid hemorrhage from middle cerebral artery-0.962 |
|  | Chronic subdural hematoma-0.908 | Cerebral infarction, unspecified-0.943 | No operation due to patient's family-0.957 |
|  | Rupture of internal carotid aneurysm-0.906 | Monoplegia of lower limb-0.942 | Subarachnoid hemorrhage from posterior communicating artery-0.957 |
|  | Unspecified injury of head-0.894 | Occlusion and stenosis of basilar artery-0.941 | Subarachnoid hemorrhage, unspecified-0.956 |
|  | Rupture of cerebral aneurysm-0.884 | Occlusion and stenosis of anterior cerebral artery-0.941 | Other subarachnoid hemorrhage-0.955 |
|  | Other rupture of cerebral aneurysm-0.883 | Visual field defects-0.940 | Rupture of anterior communicating artery aneurysm-0.953 |
|  | Ruptured of anterior communicating artery aneurysm-0.882 | Occlusion and stenosis of carotid artery-0.940 | Bronchitis, not specified as acute or chronic-0.950 |
| Laboratory tests | Cerebrospinal fluid color: red color-0.933 | Platelet aggregation test with turbidimetry: high-0.915 | Cerebrospinal fluid transparency: turbid-0.975 |
|  | Cerebrospinal fluid transparency: turbid-0.904 | Plasma protein C: high-0.914 | Cerebrospinal fluid color: blood color-0.959 |
|  | Cerebrospinal fluid color: orange-0.863 | Platelet aggregation test with turbidimetry: low-0.910 | White blood cell count in cerebrospinal fluid: high-0.958 |
|  | Sodium in arterial blood: high-0.827 | Plasma red blood cell aggregation index: high-0.908 | Cerebrospinal fluid color: red-0.957 |
|  | Chlorine content of cerebrospinal fluid: high-0.778 | Plasma red blood cell aggregation index: low-0.902 | Serum irregular antibody screening (3-cell): negative-0.948 |
|  | Cerebrospinal fluid color: yellow-0.773 | Whole blood viscosity (200): high-0.897 | Plasma activated partial thromboplastin time: low-0.920 |
|  | Cerebrospinal fluid color: light orange-0.769 | Plasma protein S: high-0.892 | Cerebrospinal fluid color: orange-0.917 |
|  | Arterial partial pressure of oxygen: high-0.758 | Whole blood viscosity (200): low-0.892 | Cerebrospinal fluid glucose: high-0.911 |
|  | Arterial oxygen saturation: high-0.750 | Total glycated hemoglobin: high-0.891 | Percentage of whole blood mononuclear cells: low-0.908 |
|  | Arterial blood lactic acid: high-0.744 | Two hours post-meal blood sugar: high-0.888 | Arterial potassium: normal-0.898 |
| Physical examinations and procedures | Embolization of intracranial aneurysm-0.985 | Percutaneous drug-eluting stent implantation-0.861 | Embolization of intracranial aneurysm-0.986 |
|  | Aneurysm clipping-0.974 | Percutaneous drug-eluting stent implantation of subclavian artery-0.848 | Aneurysm clipping-0.974 |
|  | Embolization of intracranial vessels-0.960 | Transcranial angioplasty-0.822 | Skull titanium plate placement-0.965 |
|  | Drilling and drainage under dura mater-0.954 | Percutaneous balloon angioplasty of common carotid artery-0.812 | Embolization of intracranial vessels-0.959 |
|  | Percutaneous intracranial artery stenting-0.952 | Thrombolytic agent input-0.792 | Percutaneous intracranial artery stenting-0.958 |
|  | Embolization of intracranial vessels-0.935 | Percutaneous drug-eluting stent implantation-0.746 | Autologous blood transfusion-0.954 |
|  | Percutaneous middle cerebral artery stenting-0.934 | Percutaneous balloon angioplasty-0.706 | Drilling and drainage under dura mater-0.950 |
|  | Superficial temporal middle cerebral artery bypass-0.933 | Percutaneous non drug eluting stent implantation-0.691 | Vertebral angiography-0.948 |
|  | Vascular resection of spinal cord malformation-0.931 | Internal carotid artery embolization-0.689 | Artificial Dural patch repair-0.947 |
|  | Percutaneous balloon angioplasty of middle cerebral artery-0.927 | Percutaneous balloon angioplasty of jugular vein-0.652 | Superficial temporal middle cerebral artery bypass-0.936 |
| Medications | Hypertonic sodium chloride hydroxyethyl starch 40 injection-0.938 | Probucol tablet-0.938 | Tramadol-0.987 |
|  | Nimodipine-0.895 | Songling Xuemaikang capsule ^c^ -0.924 | Fasudil -0.983 |
|  | Fructose sodium diphosphate injection-0.894 | Yufeng Ningxin Drop Pills ^c^ -0.920 | Dezocine injection-0.982 |
|  | Namefen injection-0.876 | Naoxuekang Capsule ^c^ -0.919 | Triston hydrochloride-0.981 |
|  | Fasudil-0.855 | Annao Wan ^c^ -0.918 | Fructose for injection-0.981 |
|  | Oral Rehydration Salt-0.825 | Maixuekang Capsules ^c^ -0.915 | Nalgene injection-0.981 |
|  | Oral protein hydrolysate-0.800 | Urinary kallidinogenase injection-0.909 | Papaverine-0.978 |
|  | Xingnaojing ^c^ -0.797 | Butylphthalide-0.904 | Ropivacaine Needle-0.977 |
|  | Urokinase injection-0.793 | Shugan Wan ^c^ -0.900 | Succinyl Gelatin-0.976 |
|  | Chlorpromazine-0.791 | Danxi granule ^c^ -0.897 | Neostigmine methyl sulfate-0.976 |
| Others | Neurosurgery ICU-0.924 | Allergic to dimethylbiguanide (a drug)-0.858 | ICU of Neurosurgery department-0.976 |
|  | Ventilator utilization-0.796 | Allergic to vinpocetine (a drug)-0.852 | Discharge department: Neurosurgery department-0.964 |
|  | Discharge department: Neurosurgery department-0.796 | Allergic to Iopromide (a drug)-0.852 | Admission department: Neurosurgery department-0.962 |
|  | Admission department: Neurosurgery department-0.782 | Allergic to Xueshuantong ^c^ -0.838 | Blood type B-0.960 |
|  | Allergic to meropenem (a drug)-0.777 | Allergic to compound aminophenazone (a drug)-0.836 | Blood type O-0.958 |
|  | Self-payment-0.772 | Allergic to Youweixian ^c^ -0.831 | Blood type A-0.951 |
|  | Allergic to albumin (a drug)-0.743 | Allergic to Salvia Miltiorrhiza (a drug)-0.821 | Blood type AB-0.950 |
|  | Vascular surgery ICU-0.739 | Allergic to statins (a drug)-0.820 | Rh blood type positive-0.946 |
|  | Allergic to lumbrokinase (a drug)-0.708 | Discharge department: Neurology department-0.794 | Occupation: Farmer-0.877 |
|  | Admission department: Vascular surgery department-0.702 | Allergic to butylphthalide (a drug)-0.792 | Admission department: Vascular surgery department-0.855 |

^a:^ Embedding vectors of concepts were trained with the full corpora.

^b:^ Embedding vectors of concepts were trained with the stroke corpora.

^c^: Traditional Chinese medication.

## Table S3. Differences in patient characteristics between the two clusters obtained from the clustering analysis based on the shuffled full corpora.

| Features/Concepts | Cluster 1 (n=6922) | Cluster 2 (n=1310) | P value | Features/Concepts | Cluster 1 (n=6922) | Cluster 2 (n=1310) | P value |
| --- | --- | --- | --- | --- | --- | --- | --- |
| Primary diagnosis (ischemic stroke) | 6495 (93.83%) | 340 (25.95%) | <0.001 | **Source of admission** |  |  | <0.001 |
| Age (Years) | 59.10±12.96 | 56.66±13.55 | <0.001 | Emergency department | 4526(65.39%) | 1065(81.30%) |  |
| Gender (Male) | 5107 (73.78%) | 703(53.66%) | <0.001 | Clinic | 2396(34.61%) | 245(18.70%) |  |
| Length of stay (day) | 9 (7, 12) | 9 (5, 16) | <0.001 | **Department at admission** |  |  | <0.001 |
| Cost (thousand RMB yuan) | 13.69 (10.72, 18.00) | 89.23(41.51, 157.22) | <0.001 | Neurosurgery department | 234(3.38%) | 891(68.02%) |  |
| Discharge Route |  |  | <0.001 | Vascular surgery department | 75(1.08%) | 243(18.55%) |  |
| Transfer to other hospital | 260(3.76 %) | 147(11.22%) |  | Neurology department | 6224(89.92%) | 137(10.46%) |  |
| Transfer to community hospital | 105(1.52%) | 12(0.92%) |  | Other Department at admission | 389(5.62%) | 39(2.98%) |  |
| Rehabilitation | 6487(93.72%) | 1020(77.86%) |  | **Department at discharge** |  |  | <0.001 |
| Death | 45(0.65%) | 91(6.95%) |  | Neurosurgery department | 229(3.31%) | 996(76.03%) |  |
| Other | 25(0.36%) | 40(3.05%) |  | Vascular surgery department | 69(1.00%) | 139(10.61%) |  |
| Readmission | 1308(18.90%) | 274(20.92%) | 0.089 | Neurology department | 6224(89.92%) | 137(10.46%) |  |
| Intensive care unit (ICU) |  |  | <0.001 | Department of geriatrics | 348(5.03%) | 5(0.38%) |  |
| Neurosurgery ICU | 13(0.19%) | 690(52.67%) |  | Other Department at discharge | 52(0.75%) | 33(2.52%) |  |
| Vascular surgery ICU | 68(0.98%) | 208(15.88%) |  | **Ventilator** | 105(1.52%) | 544(41.53%) | <0.001 |
| Neurology ICU | 269(3.89%) | 111(8.47%) |  | **Laboratory tests related to stroke** |  |  |  |
| Other ICU | 3(0.04%) | 16(1.22%) |  | Complete blood leukocyte count: low | 72(1.04%) | 7(0.53%) | <0.001 |
| Procedures related to stroke |  |  |  | Complete blood leukocyte count: high | 934(13.49%) | 649(49.54%) |  |
| Percutaneous angioplasty or atherectomy of precerebral (extracranial) vessel(s) | 36(0.52%) | 28(2.14%) | <0.001 | Complete blood leukocyte count: medium | 5552(80.21%) | 523(39.92%) |  |
| Percutaneous angioplasty or atherectomy of intracranial vessel(s) | 7(0.10%) | 44(3.36%) | <0.001 | Hematocrit: low | 2692(38.89%) | 659(50.31%) | <0.001 |
| Percutaneous insertion of carotid artery stent(s) | 31(0.45%) | 34(2.60%) | <0.001 | Hematocrit: high | 184(2.66%) | 22(1.68%) |  |
| Percutaneous insertion of other precerebral (extracranial) artery stent(s) | 32(0.46%) | 54(4.12%) | <0.001 | Hematocrit: medium | 3721(53.76%) | 498(38.02%) |  |
| Percutaneous insertion of intracranial vascular stent(s) | 10(0.14%) | 99(7.56%) | <0.001 | Complete blood platelet count: low | 45(0.65%) | 28(2.14%) | <0.001 |
| Other cranial puncture | 4(0.06%) | 56(4.27%) | <0.001 | Complete blood platelet count: high | 460(6.65%) | 75(5.73%) |  |
| Other craniotomy | 5(0.07%) | 111(8.47%) | <0.001 | Complete blood platelet count: medium | 6053(87.45%) | 1076(82.14%) |  |
| Incision of cerebral meninges | 2(0.03%) | 39(2.98%) | <0.001 | Plasma D-dimer: high | 3604(52.07%) | 706(53.89%) | <0.001 |
| Other incision of brain | 10(0.14%) | 162(12.37%) | <0.001 | Plasma D-dimer: medium | 1761(25.44%) | 54(4.12%) |  |
| Other excision of vessel | 0(0.00%) | 21(1.60%) | <0.001 | Plasma fibrinogen: low | 137(1.98%) | 40(3.05%) | 0.006 |
| Other surgical occlusion of vessels | 0(0.00%) | 13(0.99%) | <0.001 | Plasma fibrinogen: high | 1653(23.88%) | 336(25.65%) |  |
| Extracranial-intracranial (EC-IC) vascular bypass | 0(0.00%) | 37(2.82%) | <0.001 | Plasma fibrinogen: medium | 4754(68.68%) | 841(64.20%) |  |
| Clipping of aneurysm | 0(0.00%) | 211(16.11%) | <0.001 | Serum albumin: low | 3528(50.97%) | 646(49.31%) | 0.023 |
| Endovascular repair or occlusion of head and neck vessels | 2(0.03%) | 330(25.19%) | <0.001 | Serum albumin: high | 0(0.00%) | 1(0.08%) |  |
| Endovascular removal of obstruction from head and neck vessel(s) | 39(0.56%) | 57(4.35%) | <0.001 | Serum albumin: medium | 3058(44.18%) | 615(46.95%) |  |
| Injection or infusion of thrombolytic agent | 344(4.97%) | 61(4.66%) | 0.681 | Serum interleukin 6: high | 183(2.64%) | 157(11.98%) | <0.001 |
| Disease diagnoses occurred in at least 3% stroke patients |  |  |  | Serum interleukin 6: medium | 245(3.54%) | 24(1.83%) |  |
| Acute cerebrovascular disease | 6803(98.28%) | 1272(97.10%) | 0.006 | Serum high-sensitivity C-reactive protein: high | 628(9.07%) | 51(3.89%) | <0.001 |
| Essential hypertension | 4616(66.69%) | 588(44.89%) | <0.001 | Serum high-sensitivity C-reactive protein: medium | 322(4.65%) | 26(1.98%) |  |
| Other nervous system disorders | 3845(55.55%) | 256(19.54%) | <0.001 | Serum low density lipoprotein: high | 1111(16.05%) | 232(17.71%) | <0.001 |
| Paralysis | 3735(53.96%) | 119(9.08%) | <0.001 | Serum low density lipoprotein: medium | 5476(79.11%) | 791(60.38%) |  |
| Occlusion or stenosis of precerebral arteries | 3317(47.92%) | 181(13.82%) | <0.001 | Serum triglyceride: high | 2135(30.84%) | 302(23.05%) | <0.001 |
| Disorders of lipid metabolism | 2452(35.42%) | 59(4.50%) | <0.001 | Serum triglyceride: medium | 4479(64.71%) | 961(73.36%) |  |
| Diabetes mellitus without complication | 1908(27.56%) | 142(10.84%) | <0.001 | Serum high density lipoprotein: low | 1951(28.19%) | 193(14.73%) | <0.001 |
| Other & ill-defined cerebrovascular disease | 1300(18.78%) | 152(11.60%) | <0.001 | Serum high density lipoprotein: high | 977(14.11%) | 342(26.11%) |  |
| Coronary atherosclerosis & other heart disease | 1004(14.50%) | 107(8.17%) | <0.001 | Serum high density lipoprotein: medium | 3659(52.86%) | 488(37.25%) |  |
| Attention-deficit conduct and disruptive behavior disorders | 811(11.72%) | 33(2.52%) | <0.001 | Serum potassium: low | 796(11.50%) | 206(15.73%) | <0.001 |
| Late effects of cerebrovascular disease | 741(10.70%) | 56(4.27%) | <0.001 | Serum potassium: high | 16(0.23%) | 17(1.30%) |  |
| Diabetes mellitus with complications | 478(6.91%) | 26(1.98%) | <0.001 | Serum potassium: medium | 5814(83.99%) | 1042(79.54%) |  |
| Nutritional deficiencies | 468(6.76%) | 3(0.23%) | <0.001 | Serum sodium: low | 559(8.08%) | 288(21.98%) | <0.001 |
| Peripheral & visceral atherosclerosis | 444(6.41%) | 6(0.46%) | <0.001 | Serum sodium: high | 56(0.81%) | 68(5.19%) |  |
| Thyroid disorders | 269(3.89%) | 9(0.69%) | <0.001 | Serum sodium: medium | 6011(86.84%) | 909(69.39%) |  |
| Fluid & electrolyte disorders | 555(8.02%) | 270(20.61%) | <0.001 | Serum glucose: low | 82(1.18%) | 10(0.76%) | <0.001 |
| Other lower respiratory disease | 403(5.82%) | 224(17.10%) | <0.001 | Serum glucose: high | 2508(36.23%) | 862(65.80%) |  |
| Pneumonia | 318(4.59%) | 189(14.43%) | <0.001 | Serum glucose: medium | 4078(58.91%) | 394(30.08%) |  |
| Chronic obstructive pulmonary disease and bronchiectasis | 218(3.15%) | 145(11.07%) | <0.001 | Serum homocysteine: high | 2513(36.30%) | 73(5.57%) | <0.001 |
| Deficiency and other anemia | 169(2.44%) | 160(12.21%) | <0.001 | Serum homocysteine: medium | 3819(55.17%) | 147(11.22%) |  |
| Aortic, peripheral & visceral artery aneurysms | 178(2.57%) | 89(6.79%) | <0.001 | Serum total cholesterol: low | 5598(80.87%) | 1023(78.09%) | 0.002 |
| Coma, stupor & brain damage | 161(2.33%) | 103(7.86%) | <0.001 | Serum total cholesterol: high | 230(3.32%) | 64(4.89%) |  |
| Congestive heart failure, non-hypertension | 182(2.63%) | 66(5.04%) | <0.001 | Serum total cholesterol: medium | 786(11.36%) | 176(13.44%) |  |

## Figure S1. An illustration of the proposed embedding-based representation and two reference representations at the feature and patient levels using simple examples. Suppose there were two patients with discrete and continuous features in the corpus. The continuous features were discretized according to the predetermined standards. Three representations of features were the embedding vectors trained by the Skip-gram algorithm, the one-hot codes whose length was the number of the discrete features, and the raw representations (the one-hot codes of the discrete features or raw values of the continuous features). Embedding-based patient representations were the average of the embedding vectors of the features. The other two patient representations were the multi-hot representation (bitwise summations of the one-hot codes) and mixture representation.

##
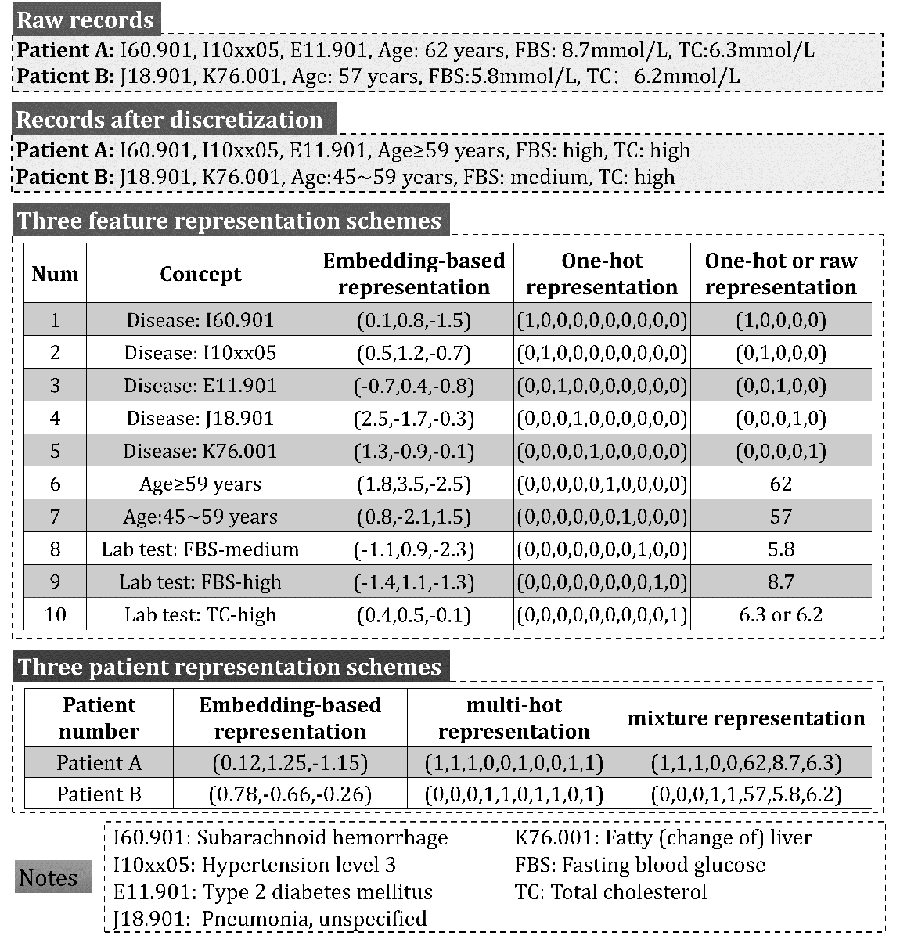


## Figure S2. Line chart of Silhouette index changing with the cluster number in k-means clustering analysis. The shuf_FC-based representation, the shuf_SC-based representation, the init_FC-based representation and the init_SC-based representation were trained with the shuffled full corpora, the shuffled stroke corpora, the initial full corpus and the initial stroke corpus by the Skip-gram algorithm (window size = 5), respectively. And the maxwin_FC-based representation and the maxwin_SC-based representation were trained with the full corpora and full corpora by the Skip-gram algorithm with the maximum window size (window size = 224 and 255), respectively. The abbreviations of the representations (e.g., shuf_FC_based representation, inint_SC-based representation) were only used in the appendixes, and they were described as full names in the manuscript.


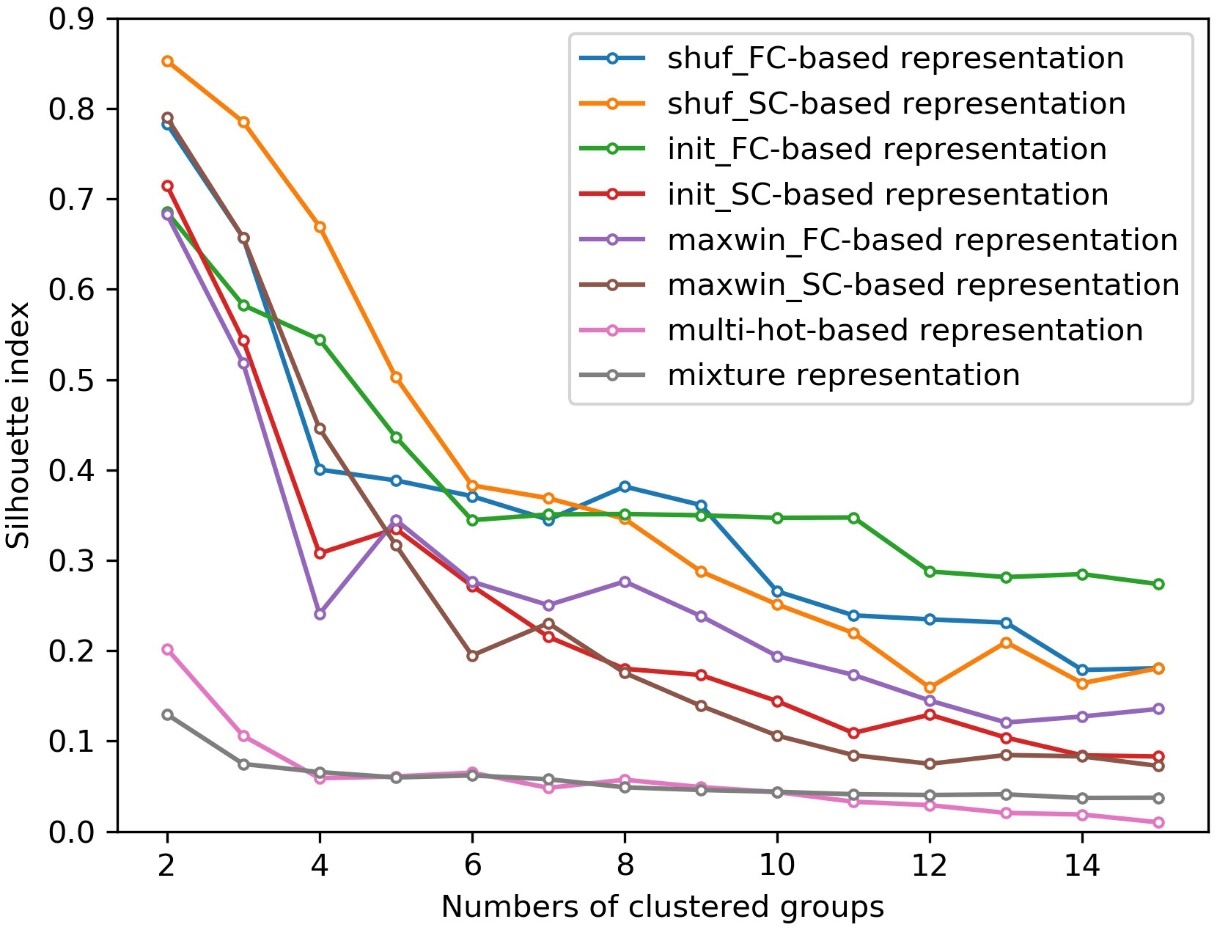

Supplement: Multimedia Appendix 1 [file medinform_v9i7e19905_app1.docx]
